# Crystal structures of two bis-carbamoyl­methyl­phosphine oxide (CMPO) compounds

**DOI:** 10.1107/S205698901900820X

**Published:** 2019-06-14

**Authors:** Andrew I. VanderWeide, Richard J. Staples, Shannon M. Biros

**Affiliations:** a Department of Chemistry, Grand Valley State University, 1 Campus Dr., Allendale, MI 49401, USA; b Center for Crystallographic Research, Department of Chemistry, Michigan State University, East Lansing, MI 48824, USA

**Keywords:** crystal structure, carbamoyl­methyl­phosphine oxide, multidentate ligand, hydrogen bonds, C—H⋯π inter­actions, π–π stacking inter­actions

## Abstract

Two bis-carbamoyl­methyl­phosphine oxide compounds, namely {[(3-{[2-(di­phen­yl­phosphino­yl)ethanamido]­meth­yl}benz­yl)carbamo­yl]meth­yl}di­phenyl­phos­phine oxide, C_36_H_34_N_2_O_4_P_2_, (I), and diethyl [({2-[2-(di­eth­oxy­phosphino­yl)ethanamido]­eth­yl}carbamo­yl)meth­yl]phospho­nate, C_14_H_30_N_2_O_8_P_2_, (II), were synthesized *via* nucleophilic acyl substitution reactions between an ester and a primary amine. Hydrogen-bonding inter­actions are present in both crystals, but these inter­actions are intra­molecular in the case of compound (I) and inter­molecular in compound (II). Intra­molecular π–π stacking inter­actions are also present in the crystal of compound (I) with a centroid–centroid distance of 3.9479 (12) Å and a dihedral angle of 9.56 (12)°. Inter­molecular C—H⋯π inter­actions [C⋯centroid distance of 3.622 (2) Å, C—H⋯centroid angle of 146°] give rise to supra­molecular sheets that lie in the *ab* plane. Key geometric features for compound (I) involve a nearly planar, *trans-*amide group with a C—N—C—C torsion angle of 169.12 (17)°, and a torsion angle of −108.39 (15)° between the phosphine oxide phospho­rus atom and the amide nitro­gen atom. For compound (II), the electron density corresponding to the phosphoryl group was disordered, and was modeled as two parts with a 0.7387 (19):0.2613 (19) occupancy ratio. Compound (II) also boasts a *trans*-amide group that approaches planarity with a C—N—C—C torsion angle of −176.50 (16)°. The hydrogen bonds in this structure are inter­molecular, with a *D*⋯*A* distance of 2.883 (2) Å and a *D*—H⋯*A* angle of 175.0 (18)° between the amide hydrogen atom and the P=O oxygen atom. These non-covalent inter­actions create ribbons that run along the *b*-axis direction.

## Abstract 2

The crystal structures of two multidentate CMPO-containing organic ligands are described. Both compounds feature N—H⋯O hydrogen bonds in the solid state.

## Chemical context   

The carbamoyl­methyl­phosphine oxide (CMPO) moiety has found use as the chelating portion of a ligand in the TRUEX process for the remediation of nuclear waste (Horwitz *et al.*, 1985 ▸). It has been shown that the CMPO group binds lanthanide (*Ln*) and actinide (*An*) metals in a 1:2 or 1:3 metal-ligand ratio in solution, depending on the size of the metal ion. Many researchers have attempted to mimic this solution stoichiometry by tethering two, three or four CMPO groups together *via* an organic scaffold (Dam *et al.*, 2007 ▸; Leoncini *et al.*, 2017 ▸; Miyazaki *et al.*, 2015 ▸; Sharova *et al.*, 2014 ▸; Werner & Biros, 2019 ▸). In some cases, these multidentate ligands have demonstrated an increased binding affinity for certain *Ln* and *An* ions, as well as an increased ability to extract these metals out of aqueous solutions. To this end, we report here the synthesis of compounds (I) and (II) and their characterization by ^1^H, ^13^C, and ^31^P NMR spectroscopy, and by X-ray crystallography.
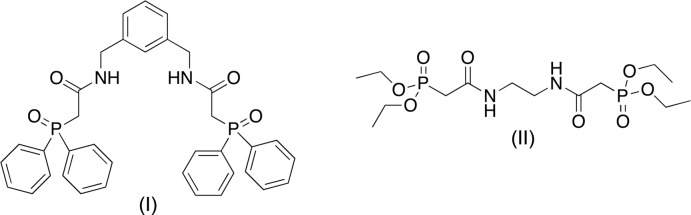


## Structural commentary   

**Figure 1:**
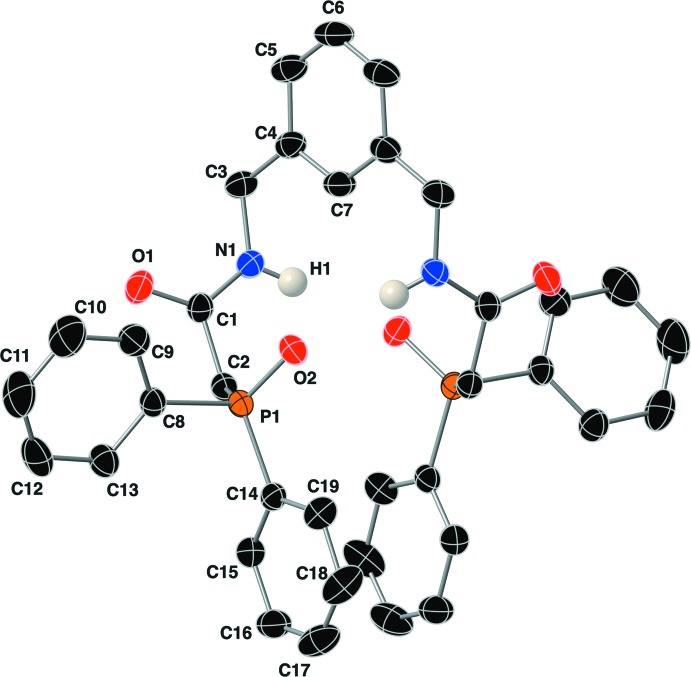
The complete mol­ecular structure of compound (I), with the atom-labeling scheme. Unlabeled atoms are related to labeled atoms by the crystallographic twofold axis. Displacement ellipsoids are drawn at the 50% probability level, and hydrogen atoms bonded to carbon atoms have been omitted for clarity.

The structure of compound (I) was solved in the monoclinic space group *C*2/*c*. Since the entire mol­ecule straddles a twofold symmetry axis, the asymmetric unit is composed of one half of the compound. The complete mol­ecular structure of compound (I) is shown in Fig. 1 ▸ along with the atom-labeling scheme. The P=O bond length is 1.4915 (13) Å, with P—C bond lengths that range from 1.7988 (18) to 1.8169 (19) Å. The τ_4_ descriptor for fourfold coordination around the phospho­rus atom P1 is 0.95, indicating a nearly perfect tetra­hedral geometry of the phosphine oxide group (where 0.00 = square-planar, 0.85 = trigonal–pyramidal, and 1.00 = tetra­hedral; Yang *et al.*, 2007 ▸). The geometry between the amide nitro­gen atom N1 and the β-phosphine oxide phospho­rus atom P1 is defined by a P1—C2—C1—N1 torsion angle of −108.39 (15)°. The amide group adopts a nearly perfect *trans* geometry with a C3—N1—C1—C2 torsion angle of 169.12 (17)°, and is staggered with respect to the plane of the C4–C7 aromatic ring with a H1—N1—C3—C4 torsion angle of 59.1 (17)°.

Intra­molecular non-covalent inter­actions are also present in the crystal of compound (I). Hydrogen bonds between the amide hydrogen H1 and the phosphine oxide oxygen atom O2(−*x* + 1, *y*, −*z* + 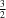) have a *D*⋯*A* distance of 2.940 (2) Å and a *D*—H⋯*A* angle of 168 (2)° (Fig. 3 ▸ and Table 1 ▸). The C14–C19 aromatic ring of this compound is engaged in an intra­molecular π–π stacking inter­action with its symmetry-derived counterpart with an inter­centroid distance of 3.9479 (12) Å, slippage of 1.521 (1) Å and a dihedral angle of 9.56 (12)°.

**Figure 2:**
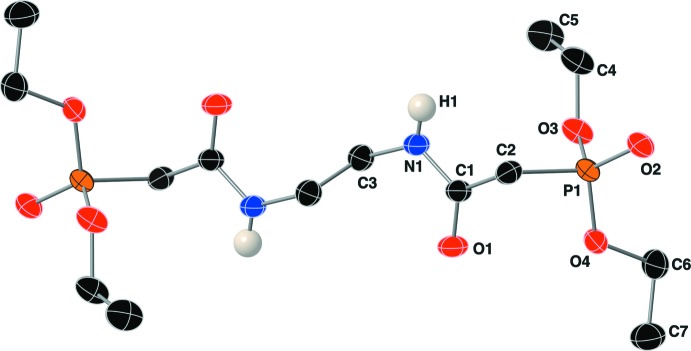
The mol­ecular structure of compound (II), with the atom-labeling scheme. Unlabeled atoms are related to labeled atoms by a crystallographic inversion center. Displacement ellipsoids are drawn at the 50% probability level, only the major component and hydrogen atoms bonded to nitro­gen atoms have been included for clarity.

**Figure 3:**
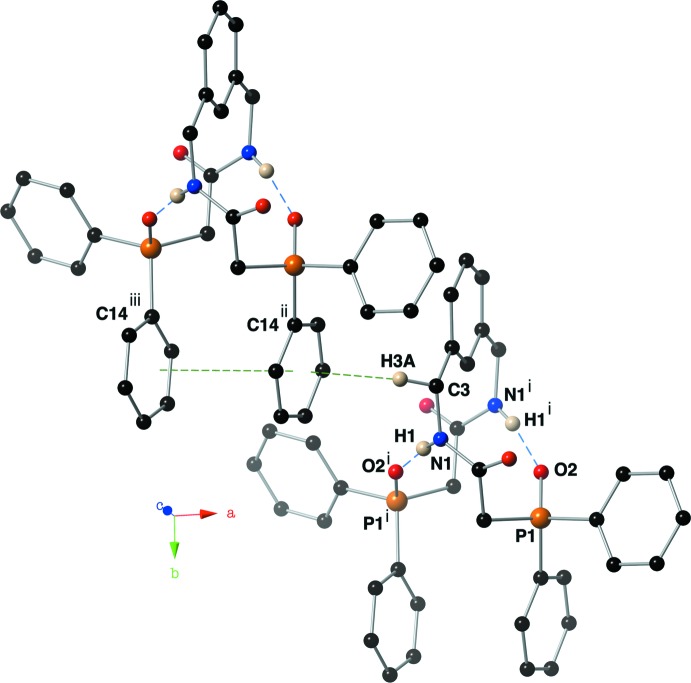
Depiction of non-covalent inter­actions present in the crystal of compound (I) using a ball-and-stick model with standard CPK colors. Intra­molecular hydrogen bonds are shown as blue dashed lines; intra­molecular π–π and inter­molecular C—H⋯π inter­actions are shown with green dashed lines. Symmetry codes: (i) 1 − *x*, *y*, 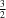 − *z*; (ii) −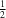 + *x*, 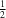 + *y*, *z*; (iii) 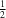 − *x*, 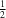 + *y*, 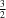 − *z*.

Compound (II) crystallizes in the ortho­rhom­bic space group *Pbca*. Since the mol­ecule lies on an inversion center (at 2 − *x*, 1 − *y*, 1 − *z*), the asymmetric unit comprises one half of the mol­ecule. The electron density corresponding to the atoms of the phosphoryl group was disordered and was modeled over two positions with a 0.7387 (19):0.2613 (19) occupancy ratio (see the *Refinement* section for more details). The complete mol­ecular structure of the major component of compound (II) is shown in Fig. 2 ▸ along with the labeling scheme. For the major component, the P=O bond length is 1.474 (2) Å, with P—O bond lengths of 1.5791 (16) and 1.5619 (15) Å, and a P—C bond length of 1.801 (2) Å. The τ_4_ descriptor for fourfold coordination around the phospho­rus atom of the major component, P1, is 0.93, indicating that the geometry of the phosphoryl group is slightly distorted from an ideal tetra­hedron. The geometry between the amide nitro­gen atom N1 and the β-phosphoryl group phospho­rus atom P1 is defined by a N1—C1—C2—P1 torsion angle of −111.8 (2)°. The amide group of this compound also adopts a nearly perfect *trans* geometry with a C3—N1—C1—C2 torsion angle of −176.50 (16)°.

## Supra­molecular features   

**Figure 4:**
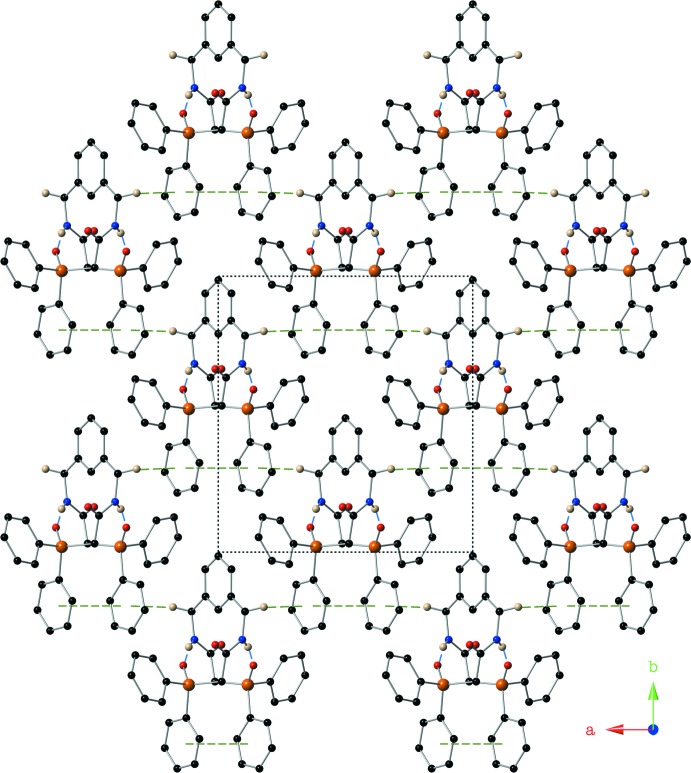
A view down the *c*-axis of compound (I) showing the supra­molecular sheets that are held together with intra­molecular C—H⋯π inter­actions using a ball-and-stick model with standard CPK colors. Hydrogen bonds are depicted with blue dashed lines, while π–π and C—H⋯π inter­actions are shown with green dashed lines. Only (N)H1 and (C)H3*A* are shown for clarity.

The C14–C19 aromatic ring of compound (I) hosts a C—H⋯π inter­action with H3*A* (symmetry code: −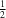 + *x*, 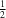 + *y*, *z*) with a C⋯centroid distance of 3.622 (2) Å and a C—H⋯centroid angle of 146°. These non-covalent inter­actions create supra­molecular sheets of compound (I) that lie in the *ab* plane (Fig. 4 ▸).

**Figure 5:**
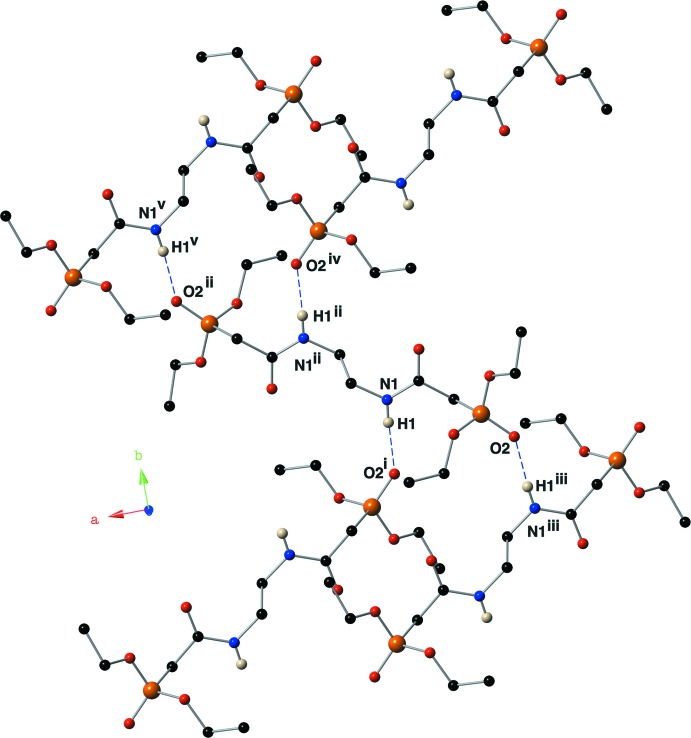
Depiction of the hydrogen-bonding network present in the crystal of compound (II) using a ball-and-stick model with standard CPK colors. The minor component of the disordered phosphoryl group is omitted for clarity. Inter­molecular hydrogen bonds are shown with blue dashed lines. Symmetry codes: (i) *x* + 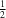, −*y* + 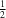, −*z* + 1; (ii) −*x* + 2, −*y*, −*z* + 1; (iii) *x* − 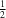, −*y* + 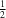, −*z* + 1; (iv) −*x* + 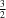, *y* − 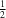, *z*; (v) −*x* + 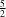, *y* − 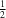, *z*.

**Figure 6:**
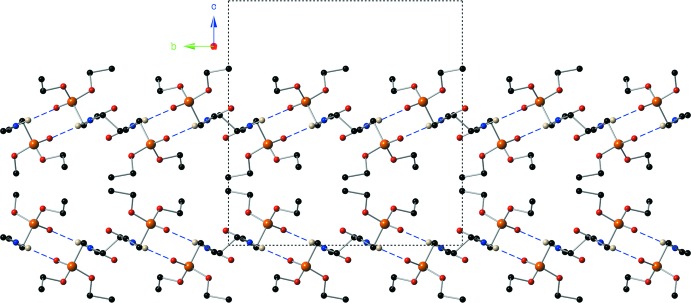
A view down the *a*-axis of the crystal of compound (II) showing the supra­molecular ribbons that are formed *via* inter­molecular hydrogen-bonding inter­actions. For clarity, only the major component of the disorder is shown.

The crystal structure of compound (II) displays inter­molecular hydrogen bonds between the amide hydrogen H1 and the oxygen atom O2 of the phosphoryl group of a neighboring mol­ecule (symmetry code: *x* + 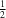, −*y* + 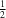, −*z* + 1; Fig. 5 ▸ and Table 2 ▸). This hydrogen bond is present for both parts of the disordered phosphoryl group. For the major component, this hydrogen bond has a *D*⋯*A* distance of 2.883 (2) Å with a *D*—H⋯*A* angle of 175.0 (18)°. This hydrogen bond forms ribbons of compound (I) that run along the *b*-axis direction (Fig. 6 ▸).

## Database survey   

The Cambridge Structural Database (CSD, Version 5.40, November 2018; Groom *et al.*, 2016 ▸) contains 19 structures which have a CMPO group as part of an organic compound. (This count excludes metal complexes.) Of these, seven structures have two or more CMPO groups tethered to one another *via* an organic scaffold. The most similar structures to compound (I) are CIWFAR (Ouizem *et al.*, 2014 ▸) and SISLIQ (Artyushin *et al.*, 2006 ▸). Both structures use an aromatic ring as the scaffold to present two phenyl-substituted CMPO groups. In SISLIQ, a 1,2-disubstituted benzene ring is utilized to present the CMPO groups. In CIWFAR, the scaffold is a pyridine ring where the 2- and 6-positions bear CMPO groups, which makes it directly analogous to compound (I). The amide hydrogens of CIWFAR are engaged in inter­molecular hydrogen bonds with the oxygen atoms of the phosphine oxide groups [rather than the intra­molecular inter­action observed for compound (I)], and the pyridine nitro­gen is hydrogen bonded to the –OH group of a solvent methanol mol­ecule. The hydrogen atoms of the pyridine scaffold inter­act with the phenyl rings of the phosphine oxide *via* inter­molecular C—H⋯π inter­actions. A structure closely related to compound (II) was reported by the Rebek group as OGIVIJ (Amrhein, *et al.*, 2002 ▸). Here, a resorcin[4]arene scaffold presents two eth­oxy-substituted CMPO units. We also note that the structure of compound (II) complexed with Sm(NO_3_)_3_ has been reported in this journal (Stoscup *et al.*, 2014 ▸).

## Synthesis and crystallization   

**Compound (I):** 1,3-Bis(amino­meth­yl)benzene (128 mg, 0.124 mL, 0.785 mmol) and the *p*-nitro­phenyl ester of di­phenyl­phosphono­acetate (Arnaud-Neu *et al.*, 1996 ▸) (1.0 g, 3.14 mmol) were dissolved in anhydrous, ethanol-free chloro­form (30 mL). The solution was heated to 313 K and stirred for three days. The reaction mixture was then allowed to cool to room temperature, a small amount of 40% KOH was added (*ca.* 3 mL) and the solution was stirred for 3.5 h. The organic layer was separated, washed with brine (3 × 10 mL), dried over solid magnesium sulfate and concentrated under reduced pressure. The crude product was triturated multiple times with ethyl acetate to give a white solid in 91% yield. X-ray quality crystals of compound (I) were grown by slow evaporation of a chloro­form solution. ^1^H NMR (400 MHz, CDCl_3_): δ 7.91 (*t*, *J* = 5.3 Hz, 2H, –N*H*), 7.7–7.3 (*m*, 20H), 7.1–6.8 (*m*, 4H), 4.24 (*d*, *J* = 7.2 Hz, 4H), 3.36 (*d*, *J*_P–H_ = 13.2 Hz, 4H); ^13^C NMR (100 MHz, CDCl_3_): δ 164.7 (*d*, *J*_P–C_ = 4.5 Hz), 138.3, 132.5, 131.9, 131.2–130.5 (*broad*), 129.5–128.3 (*broad*), 126.9–126.1 (*broad*), 43.5, 38.6; ^31^P NMR (161 MHz, CDCl_3_): δ 30.6.

**Compound (II):** Ethyl­ene di­amine (1.0 mL, 14.9 mmol) was dissolved in 8.3 mL of methanol. The solution was cooled to 195 K, and triethyl phosphono­acetate (8.8 mL, 44.8 mmol) was added dropwise. The reaction mixture was allowed to warm to room temperature and stirred overnight. The product precipitated from the solution, was isolated by vacuum filtration and rinsed with ethyl acetate. Some of this solid was crystalline and suitable for analysis by X-ray diffraction. The remainder of the isolated product was purified by silica gel chromatography (10:1 di­chloro­methane–methanol) to give compound (II) as a white solid (37% yield). ^1^H NMR (300 MHz, CDCl_3_): δ 7.75 (*broad*, 2H, –N*H*), 4.15 (*q*, *J* = 7.0 Hz, 8H), 3.34 (*d*, *J* = 5.9 Hz, 8H), 2.85 (*q*, *J*_P–H_ = 15.8 Hz, 8H), 1.33 (*t*, *J* = 7.0 Hz, 12H); ^13^C NMR (75 MHz, CDCl_3_): δ 165.4, 62.9, 35.8 (*d*, *J*_P–C_ = 128 Hz), 16.5; ^31^P NMR (121 MHz, CDCl_3_): δ 24.5.

## Refinement   

Crystal data, data collection and structure refinement details for both compounds are summarized in Table 3 ▸. For compounds (I) and (II), all hydrogen atoms bonded to carbon atoms were placed in calculated positions and refined as riding: C—H = 0.95–1.00 Å with *U*_iso_(H) = 1.2*U*_eq_(C) for methyl­ene groups and aromatic hydrogen atoms, and *U*_iso_(H) = 1.5*U*_eq_(C) for methyl groups. For both compounds (I) and (II), the hydrogen atoms bonded to nitro­gen atoms were located using electron-density difference maps. The disordered electron density corresponding to the phosphoryl group of compound (II) was modeled over two positions with a relative occupancy ratio of 0.7387 (19):0.2613 (19). The C5—C4 and C6—C7 bond lengths were restrained using DFIX instructions in *SHELXL* (Sheldrick, 2015 ▸) at 1.5 Å to agree with known values. Atoms of each part (P1, P1*A*, O2–O4, O2*A*–O4*A*, C2, C2*A*, C5–C7, C5*A*–C7*A*) were treated with SAME and EADP commands to produce bond lengths and angles that agree with known values, and to ensure physically reasonable displacement parameters.

## Supplementary Material

Crystal structure: contains datablock(s) I, II. DOI: 10.1107/S205698901900820X/pk2617sup1.cif

Structure factors: contains datablock(s) I. DOI: 10.1107/S205698901900820X/pk2617Isup2.hkl

Supporting information file. DOI: 10.1107/S205698901900820X/pk2617Isup4.cml

Structure factors: contains datablock(s) II. DOI: 10.1107/S205698901900820X/pk2617IIsup3.hkl

Supporting information file. DOI: 10.1107/S205698901900820X/pk2617IIsup5.cml

CCDC references: 1921486, 1921485

Additional supporting information:  crystallographic information; 3D view; checkCIF report

## Figures and Tables

**Table 1:** Hydrogen-bond geometry (Å, °) for (I) *Cg* is the centroid of the C14–C19 ring.

*D*—H⋯*A*	*D*—H	H⋯*A*	*D*⋯*A*	*D*—H⋯*A*
N1—H1⋯O2^i^	0.85 (2)	2.10 (2)	2.940 (2)	168 (2)
C3—H3*A*⋯*Cg* ^ii^	0.99	2.76	3.622 (2)	146

Symmetry codes: (i) 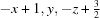; (ii) 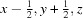.

**Table 2:** Hydrogen-bond geometry (Å, °) for (II)

*D*—H⋯*A*	*D*—H	H⋯*A*	*D*⋯*A*	*D*—H⋯*A*
N1—H1⋯O2^i^	0.832 (19)	2.05 (2)	2.883 (2)	175.0 (18)
N1—H1⋯O2*A* ^i^	0.832 (19)	1.92 (2)	2.741 (8)	170.2 (18)

Symmetry code: (i) 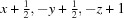.

**Table 3:** Experimental details

	(I)	(II)
Crystal data		
Chemical formula	C_36_H_34_N_2_O_4_P_2_	C_14_H_30_N_2_O_8_P_2_
*M*_r_	620.59	416.34
Crystal system, space group	Monoclinic, *C*2/*c*	Orthorhombic, *P* *b* *c* *a*
Temperature (K)	173	173
*a*, *b*, *c* (Å)	13.0352 (2), 14.1348 (4), 17.0471 (4)	8.9401 (1), 15.0535 (2), 15.7314 (3)
α, β, γ (°)	90, 90.217 (2), 90	90, 90, 90
*V* (Å^3^)	3140.90 (13)	2117.13 (5)
*Z*	4	4
Radiation type	Cu *K*α	Cu *K*α
μ (mm^−1^)	1.60	2.23
Crystal size (mm)	0.38 × 0.11 × 0.08	0.34 × 0.23 × 0.06

Data collection		
Diffractometer	Bruker APEXII CCD	Bruker APEXII CCD
Absorption correction	Multi-scan (*SADABS*; Bruker, 2013 ▸)	Multi-scan (*SADABS*; Bruker, 2013 ▸)
*T*_min_, *T*_max_	0.617, 0.754	0.612, 0.754
No. of measured, independent and observed [*I* > 2σ(*I*)] reflections	16825, 3022, 2543	10282, 2057, 1839
*R*_int_	0.050	0.028
(sin θ/λ)_max_ (Å^−1^)	0.617	0.617

Refinement		
*R*[*F* ^2^ > 2σ(*F* ^2^)], *wR*(*F* ^2^), *S*	0.041, 0.117, 1.03	0.035, 0.093, 1.05
No. of reflections	3022	2057
No. of parameters	204	154
No. of restraints	0	20
H-atom treatment	H atoms treated by a mixture of independent and constrained refinement	H atoms treated by a mixture of independent and constrained refinement
Δρ_max_, Δρ_min_ (e Å^−3^)	0.33, −0.27	0.21, −0.27

Computer programs: *APEX2* and *SAINT* (Bruker, 2013 ▸), *SHELXS* (Sheldrick, 2008 ▸), *olex2.solve* (Bourhis *et al.*, 2015 ▸), *SHELXL* (Sheldrick, 2015 ▸), *OLEX2* (Dolomanov *et al.*, 2009 ▸) and *CrystalMaker* (Palmer, 2007 ▸).

## supplementary crystallographic information

### {[(3-{[2-(Diphenylphosphinoyl)ethanamido]methyl}benzyl)carbamoyl]methyl}diphenylphosphine oxide (I) Crystal data

**Table:**

C_36_H_34_N_2_O_4_P_2_	*F*(000) = 1304
*M**_r_* = 620.59	*D*_x_ = 1.312 Mg m^−^^3^
Monoclinic, *C*2/*c*	Cu *K*α radiation, λ = 1.54178 Å
*a* = 13.0352 (2) Å	Cell parameters from 7639 reflections
*b* = 14.1348 (4) Å	θ = 4.6–71.9°
*c* = 17.0471 (4) Å	µ = 1.60 mm^−^^1^
β = 90.217 (2)°	*T* = 173 K
*V* = 3140.90 (13) Å^3^	Needle, colourless
*Z* = 4	0.38 × 0.11 × 0.08 mm

### {[(3-{[2-(Diphenylphosphinoyl)ethanamido]methyl}benzyl)carbamoyl]methyl}diphenylphosphine oxide (I) Data collection

**Table:**

Bruker APEXII CCD diffractometer	2543 reflections with *I* > 2σ(*I*)
φ and ω scans	*R*_int_ = 0.050
Absorption correction: multi-scan (SADABS; Bruker, 2013)	θ_max_ = 72.1°, θ_min_ = 4.6°
*T*_min_ = 0.617, *T*_max_ = 0.754	*h* = −16→16
16825 measured reflections	*k* = −17→16
3022 independent reflections	*l* = −21→20

### {[(3-{[2-(Diphenylphosphinoyl)ethanamido]methyl}benzyl)carbamoyl]methyl}diphenylphosphine oxide (I) Refinement

**Table:**

Refinement on *F*^2^	Primary atom site location: structure-invariant direct methods
Least-squares matrix: full	Hydrogen site location: mixed
*R*[*F*^2^ > 2σ(*F*^2^)] = 0.041	H atoms treated by a mixture of independent and constrained refinement
*wR*(*F*^2^) = 0.117	*w* = 1/[σ^2^(*F*_o_^2^) + (0.071*P*)^2^ + 1.6884*P*] where *P* = (*F*_o_^2^ + 2*F*_c_^2^)/3
*S* = 1.03	(Δ/σ)_max_ = 0.001
3022 reflections	Δρ_max_ = 0.33 e Å^−^^3^
204 parameters	Δρ_min_ = −0.27 e Å^−^^3^
0 restraints	

### {[(3-{[2-(Diphenylphosphinoyl)ethanamido]methyl}benzyl)carbamoyl]methyl}diphenylphosphine oxide (I) Special details

**Table:**

Geometry. All esds (except the esd in the dihedral angle between two l.s. planes) are estimated using the full covariance matrix. The cell esds are taken into account individually in the estimation of esds in distances, angles and torsion angles; correlations between esds in cell parameters are only used when they are defined by crystal symmetry. An approximate (isotropic) treatment of cell esds is used for estimating esds involving l.s. planes.

### {[(3-{[2-(Diphenylphosphinoyl)ethanamido]methyl}benzyl)carbamoyl]methyl}diphenylphosphine oxide (I) Fractional atomic coordinates and isotropic or equivalent isotropic displacement parameters (Å^2^)

**Table:**

	*x*	*y*	*z*	*U*_iso_*/*U*_eq_	
P1	0.61558 (3)	0.01942 (3)	0.65310 (2)	0.02456 (15)	
O1	0.51178 (11)	0.16409 (10)	0.52941 (8)	0.0387 (4)	
O2	0.63820 (10)	0.08953 (9)	0.71622 (8)	0.0319 (3)	
N1	0.40562 (13)	0.18290 (11)	0.63349 (10)	0.0304 (4)	
H1	0.3839 (17)	0.1569 (16)	0.6753 (14)	0.031 (6)*	
C1	0.46922 (13)	0.13271 (13)	0.58793 (10)	0.0279 (4)	
C2	0.48641 (14)	0.03112 (13)	0.61386 (10)	0.0279 (4)	
H2A	0.477236	−0.012087	0.568671	0.033*	
H2B	0.435750	0.013809	0.654532	0.033*	
C3	0.39481 (17)	0.28492 (14)	0.62513 (12)	0.0367 (5)	
H3A	0.321015	0.301452	0.625674	0.044*	
H3B	0.422971	0.304441	0.573764	0.044*	
C4	0.44947 (14)	0.33936 (13)	0.68985 (12)	0.0323 (4)	
C5	0.44880 (15)	0.43806 (14)	0.69115 (14)	0.0388 (5)	
H5	0.413150	0.472302	0.651594	0.047*	
C6	0.500000	0.4858 (2)	0.750000	0.0418 (7)	
H6	0.500001	0.553010	0.750000	0.050*	
C7	0.500000	0.29186 (19)	0.750000	0.0337 (6)	
H7	0.500000	0.224650	0.750000	0.040*	
C8	0.70406 (14)	0.03028 (13)	0.57209 (10)	0.0282 (4)	
C9	0.75675 (18)	0.11496 (16)	0.56285 (13)	0.0430 (5)	
H9	0.745261	0.165338	0.598650	0.052*	
C10	0.8259 (2)	0.12686 (19)	0.50205 (16)	0.0560 (7)	
H10	0.862193	0.184740	0.496512	0.067*	
C11	0.84148 (19)	0.0541 (2)	0.44976 (15)	0.0575 (7)	
H11	0.888183	0.062054	0.407633	0.069*	
C12	0.78956 (18)	−0.03057 (19)	0.45821 (14)	0.0495 (6)	
H12	0.800717	−0.080434	0.421862	0.059*	
C13	0.72129 (15)	−0.04301 (15)	0.51949 (12)	0.0355 (4)	
H13	0.686365	−0.101540	0.525499	0.043*	
C14	0.62569 (14)	−0.10082 (13)	0.68680 (11)	0.0278 (4)	
C15	0.57441 (16)	−0.17625 (14)	0.65110 (12)	0.0342 (4)	
H15	0.527354	−0.164934	0.609453	0.041*	
C16	0.59268 (17)	−0.26772 (15)	0.67688 (13)	0.0407 (5)	
H16	0.557730	−0.319191	0.652892	0.049*	
C17	0.66130 (17)	−0.28459 (16)	0.73716 (16)	0.0472 (6)	
H17	0.674521	−0.347566	0.753841	0.057*	
C18	0.71074 (17)	−0.20969 (18)	0.77322 (15)	0.0481 (6)	
H18	0.757181	−0.221448	0.815181	0.058*	
C19	0.69321 (15)	−0.11785 (15)	0.74878 (12)	0.0359 (4)	
H19	0.726984	−0.066640	0.774090	0.043*	

### {[(3-{[2-(Diphenylphosphinoyl)ethanamido]methyl}benzyl)carbamoyl]methyl}diphenylphosphine oxide (I) Atomic displacement parameters (Å^2^)

**Table:**

	*U*^11^	*U*^22^	*U*^33^	*U*^12^	*U*^13^	*U*^23^
P1	0.0270 (2)	0.0245 (2)	0.0222 (2)	0.00211 (17)	0.00302 (17)	−0.00095 (16)
O1	0.0434 (8)	0.0417 (8)	0.0310 (7)	0.0046 (6)	0.0073 (6)	0.0093 (6)
O2	0.0351 (7)	0.0325 (7)	0.0281 (6)	0.0011 (6)	0.0016 (5)	−0.0055 (5)
N1	0.0345 (8)	0.0285 (8)	0.0283 (8)	0.0040 (7)	0.0030 (7)	0.0035 (6)
C1	0.0282 (9)	0.0317 (10)	0.0238 (9)	0.0006 (7)	−0.0023 (7)	0.0012 (7)
C2	0.0294 (9)	0.0291 (9)	0.0251 (8)	0.0009 (7)	0.0022 (7)	−0.0011 (7)
C3	0.0426 (11)	0.0291 (10)	0.0385 (11)	0.0092 (8)	0.0008 (9)	0.0063 (8)
C4	0.0296 (9)	0.0283 (9)	0.0392 (10)	0.0032 (7)	0.0094 (8)	0.0039 (8)
C5	0.0317 (10)	0.0297 (10)	0.0549 (13)	0.0048 (8)	0.0128 (9)	0.0078 (9)
C6	0.0384 (15)	0.0224 (13)	0.065 (2)	0.000	0.0157 (14)	0.000
C7	0.0376 (14)	0.0230 (12)	0.0406 (15)	0.000	0.0051 (12)	0.000
C8	0.0262 (8)	0.0327 (9)	0.0256 (8)	0.0033 (7)	0.0025 (7)	0.0008 (7)
C9	0.0521 (13)	0.0356 (11)	0.0415 (12)	−0.0065 (10)	0.0124 (10)	−0.0018 (9)
C10	0.0556 (14)	0.0547 (15)	0.0577 (15)	−0.0170 (12)	0.0174 (12)	0.0078 (12)
C11	0.0456 (13)	0.0782 (18)	0.0488 (14)	−0.0083 (13)	0.0236 (11)	0.0011 (13)
C12	0.0458 (13)	0.0615 (15)	0.0414 (12)	0.0029 (11)	0.0146 (10)	−0.0137 (11)
C13	0.0328 (10)	0.0391 (11)	0.0346 (10)	−0.0002 (8)	0.0049 (8)	−0.0050 (8)
C14	0.0293 (9)	0.0271 (9)	0.0269 (8)	0.0046 (7)	0.0069 (7)	0.0024 (7)
C15	0.0392 (10)	0.0327 (10)	0.0308 (9)	0.0015 (8)	0.0041 (8)	0.0007 (8)
C16	0.0451 (11)	0.0284 (10)	0.0487 (12)	−0.0008 (9)	0.0110 (10)	0.0017 (9)
C17	0.0375 (11)	0.0345 (11)	0.0698 (16)	0.0057 (9)	0.0081 (11)	0.0192 (11)
C18	0.0340 (11)	0.0506 (13)	0.0595 (14)	0.0032 (10)	−0.0052 (10)	0.0233 (12)
C19	0.0299 (9)	0.0381 (11)	0.0398 (11)	−0.0002 (8)	−0.0003 (8)	0.0062 (9)

### {[(3-{[2-(Diphenylphosphinoyl)ethanamido]methyl}benzyl)carbamoyl]methyl}diphenylphosphine oxide (I) Geometric parameters (Å, º)

**Table:**

P1—O2	1.4915 (13)	C8—C13	1.389 (3)
P1—C2	1.8169 (19)	C9—H9	0.9500
P1—C8	1.8091 (18)	C9—C10	1.386 (3)
P1—C14	1.7988 (18)	C10—H10	0.9500
O1—C1	1.226 (2)	C10—C11	1.377 (4)
N1—H1	0.85 (2)	C11—H11	0.9500
N1—C1	1.341 (2)	C11—C12	1.382 (4)
N1—C3	1.456 (2)	C12—H12	0.9500
C1—C2	1.519 (3)	C12—C13	1.386 (3)
C2—H2A	0.9900	C13—H13	0.9500
C2—H2B	0.9900	C14—C15	1.397 (3)
C3—H3A	0.9900	C14—C19	1.393 (3)
C3—H3B	0.9900	C15—H15	0.9500
C3—C4	1.520 (3)	C15—C16	1.386 (3)
C4—C5	1.395 (3)	C16—H16	0.9500
C4—C7	1.390 (2)	C16—C17	1.381 (3)
C5—H5	0.9500	C17—H17	0.9500
C5—C6	1.379 (3)	C17—C18	1.382 (4)
C6—H6	0.9500	C18—H18	0.9500
C7—H7	0.9500	C18—C19	1.382 (3)
C8—C9	1.389 (3)	C19—H19	0.9500
			
O2—P1—C2	112.68 (8)	C9—C8—P1	118.45 (15)
O2—P1—C8	111.68 (8)	C13—C8—P1	122.32 (15)
O2—P1—C14	112.54 (8)	C13—C8—C9	119.22 (18)
C8—P1—C2	107.69 (8)	C8—C9—H9	119.6
C14—P1—C2	105.68 (9)	C10—C9—C8	120.8 (2)
C14—P1—C8	106.13 (8)	C10—C9—H9	119.6
C1—N1—H1	117.7 (15)	C9—C10—H10	120.3
C1—N1—C3	121.81 (17)	C11—C10—C9	119.4 (2)
C3—N1—H1	118.5 (15)	C11—C10—H10	120.3
O1—C1—N1	124.21 (18)	C10—C11—H11	119.8
O1—C1—C2	120.80 (17)	C10—C11—C12	120.4 (2)
N1—C1—C2	114.98 (15)	C12—C11—H11	119.8
P1—C2—H2A	109.8	C11—C12—H12	119.9
P1—C2—H2B	109.8	C11—C12—C13	120.3 (2)
C1—C2—P1	109.19 (12)	C13—C12—H12	119.9
C1—C2—H2A	109.8	C8—C13—H13	120.1
C1—C2—H2B	109.8	C12—C13—C8	119.9 (2)
H2A—C2—H2B	108.3	C12—C13—H13	120.1
N1—C3—H3A	109.1	C15—C14—P1	123.20 (15)
N1—C3—H3B	109.1	C19—C14—P1	116.79 (15)
N1—C3—C4	112.67 (16)	C19—C14—C15	119.90 (18)
H3A—C3—H3B	107.8	C14—C15—H15	120.2
C4—C3—H3A	109.1	C16—C15—C14	119.5 (2)
C4—C3—H3B	109.1	C16—C15—H15	120.2
C5—C4—C3	120.98 (19)	C15—C16—H16	119.8
C7—C4—C3	120.69 (18)	C17—C16—C15	120.5 (2)
C7—C4—C5	118.3 (2)	C17—C16—H16	119.8
C4—C5—H5	120.1	C16—C17—H17	120.1
C6—C5—C4	119.8 (2)	C16—C17—C18	119.9 (2)
C6—C5—H5	120.1	C18—C17—H17	120.1
C5—C6—C5^i^	121.4 (3)	C17—C18—H18	119.7
C5^i^—C6—H6	119.3	C19—C18—C17	120.6 (2)
C5—C6—H6	119.3	C19—C18—H18	119.7
C4—C7—C4^i^	122.2 (3)	C14—C19—H19	120.2
C4—C7—H7	118.9	C18—C19—C14	119.6 (2)
C4^i^—C7—H7	118.9	C18—C19—H19	120.2

Symmetry code: (i) −*x*+1, *y*, −*z*+3/2.

### {[(3-{[2-(Diphenylphosphinoyl)ethanamido]methyl}benzyl)carbamoyl]methyl}diphenylphosphine oxide (I) Hydrogen-bond geometry (Å, º)

*Cg* is the centroid of the C14–C19 ring.

**Table:**

*D*—H···*A*	*D*—H	H···*A*	*D*···*A*	*D*—H···*A*
N1—H1···O2^i^	0.85 (2)	2.10 (2)	2.940 (2)	168 (2)
C3—H3*A*···*Cg*^ii^	0.99	2.76	3.622 (2)	146

Symmetry codes: (i) −*x*+1, *y*, −*z*+3/2; (ii) *x*−1/2, *y*+1/2, *z*.

### Diethyl [({2-[2-(diethoxyphosphinoyl)ethanamido]ethyl}carbamoyl)methyl]phosphonate (II) Crystal data

**Table:**

C_14_H_30_N_2_O_8_P_2_	*D*_x_ = 1.306 Mg m^−^^3^
*M**_r_* = 416.34	Cu *K*α radiation, λ = 1.54178 Å
Orthorhombic, *P**b**c**a*	Cell parameters from 6034 reflections
*a* = 8.9401 (1) Å	θ = 4.1–72.0°
*b* = 15.0535 (2) Å	µ = 2.23 mm^−^^1^
*c* = 15.7314 (3) Å	*T* = 173 K
*V* = 2117.13 (5) Å^3^	Plate, colourless
*Z* = 4	0.34 × 0.23 × 0.06 mm
*F*(000) = 888	

### Diethyl [({2-[2-(diethoxyphosphinoyl)ethanamido]ethyl}carbamoyl)methyl]phosphonate (II) Data collection

**Table:**

Bruker APEXII CCD diffractometer	1839 reflections with *I* > 2σ(*I*)
φ and ω scans	*R*_int_ = 0.028
Absorption correction: multi-scan (SADABS; Bruker, 2013)	θ_max_ = 72.2°, θ_min_ = 5.6°
*T*_min_ = 0.612, *T*_max_ = 0.754	*h* = −11→10
10282 measured reflections	*k* = −18→12
2057 independent reflections	*l* = −19→16

### Diethyl [({2-[2-(diethoxyphosphinoyl)ethanamido]ethyl}carbamoyl)methyl]phosphonate (II) Refinement

**Table:**

Refinement on *F*^2^	Primary atom site location: iterative
Least-squares matrix: full	Hydrogen site location: mixed
*R*[*F*^2^ > 2σ(*F*^2^)] = 0.035	H atoms treated by a mixture of independent and constrained refinement
*wR*(*F*^2^) = 0.093	*w* = 1/[σ^2^(*F*_o_^2^) + (0.045*P*)^2^ + 0.8277*P*] where *P* = (*F*_o_^2^ + 2*F*_c_^2^)/3
*S* = 1.05	(Δ/σ)_max_ < 0.001
2057 reflections	Δρ_max_ = 0.21 e Å^−^^3^
154 parameters	Δρ_min_ = −0.27 e Å^−^^3^
20 restraints	

### Diethyl [({2-[2-(diethoxyphosphinoyl)ethanamido]ethyl}carbamoyl)methyl]phosphonate (II) Special details

**Table:**

Geometry. All esds (except the esd in the dihedral angle between two l.s. planes) are estimated using the full covariance matrix. The cell esds are taken into account individually in the estimation of esds in distances, angles and torsion angles; correlations between esds in cell parameters are only used when they are defined by crystal symmetry. An approximate (isotropic) treatment of cell esds is used for estimating esds involving l.s. planes.

### Diethyl [({2-[2-(diethoxyphosphinoyl)ethanamido]ethyl}carbamoyl)methyl]phosphonate (II) Fractional atomic coordinates and isotropic or equivalent isotropic displacement parameters (Å^2^)

**Table:**

	*x*	*y*	*z*	*U*_iso_*/*U*_eq_	Occ. (<1)
P1	0.49865 (8)	0.16848 (6)	0.58724 (5)	0.0291 (2)	0.7387 (19)
O2	0.3858 (2)	0.23508 (12)	0.56270 (16)	0.0402 (5)	0.7387 (19)
O3	0.6214 (2)	0.20596 (15)	0.64985 (15)	0.0364 (4)	0.7387 (19)
O4	0.43649 (17)	0.08482 (10)	0.63416 (10)	0.0361 (4)	0.7387 (19)
C2	0.5970 (5)	0.1213 (3)	0.49806 (18)	0.0255 (6)	0.7387 (19)
H2A	0.526044	0.087298	0.462353	0.031*	0.7387 (19)
H2B	0.639568	0.169666	0.462907	0.031*	0.7387 (19)
C4	0.6892 (3)	0.2924 (2)	0.6313 (3)	0.0433 (7)	0.7387 (19)
H4A	0.620650	0.340728	0.648672	0.052*	0.7387 (19)
H4B	0.708400	0.297943	0.569581	0.052*	0.7387 (19)
C5	0.8336 (4)	0.2991 (3)	0.6797 (3)	0.0521 (8)	0.7387 (19)
H5A	0.901244	0.251520	0.661636	0.078*	0.7387 (19)
H5B	0.813603	0.293490	0.740726	0.078*	0.7387 (19)
H5C	0.880239	0.356862	0.668357	0.078*	0.7387 (19)
C6	0.3339 (3)	0.09235 (16)	0.70583 (18)	0.0470 (6)	0.7387 (19)
H6A	0.250321	0.132632	0.691026	0.056*	0.7387 (19)
H6B	0.386886	0.117380	0.755628	0.056*	0.7387 (19)
C7	0.2754 (9)	0.0033 (4)	0.7266 (5)	0.0514 (10)	0.7387 (19)
H7A	0.209468	−0.016933	0.680694	0.077*	0.7387 (19)
H7B	0.218830	0.006108	0.779863	0.077*	0.7387 (19)
H7C	0.358860	−0.038321	0.732867	0.077*	0.7387 (19)
O1	0.69589 (11)	−0.01159 (7)	0.56001 (7)	0.0369 (3)	
N1	0.85841 (14)	0.09068 (9)	0.51170 (9)	0.0333 (3)	
C1	0.72039 (15)	0.06112 (9)	0.52745 (9)	0.0287 (3)	
C3	0.98811 (16)	0.03699 (10)	0.53232 (10)	0.0348 (3)	
H3A	0.974766	0.010625	0.589473	0.042*	
H3B	1.078035	0.075326	0.533945	0.042*	
P1A	0.5246 (3)	0.19107 (16)	0.60724 (17)	0.0291 (2)	0.2613 (19)
O2A	0.4150 (8)	0.2586 (4)	0.5806 (5)	0.0402 (5)	0.2613 (19)
O3A	0.6639 (8)	0.2236 (5)	0.6594 (5)	0.0364 (4)	0.2613 (19)
O4A	0.4656 (5)	0.1195 (3)	0.6726 (3)	0.0361 (4)	0.2613 (19)
C2A	0.5883 (17)	0.1303 (10)	0.5188 (7)	0.0255 (6)	0.2613 (19)
H2AA	0.501156	0.097716	0.495619	0.031*	0.2613 (19)
H2AB	0.618606	0.173979	0.474973	0.031*	0.2613 (19)
C4A	0.7429 (11)	0.3020 (7)	0.6334 (9)	0.0433 (7)	0.2613 (19)
H4AA	0.678295	0.354818	0.640873	0.052*	0.2613 (19)
H4AB	0.770059	0.297421	0.572537	0.052*	0.2613 (19)
C5A	0.8806 (14)	0.3114 (11)	0.6859 (11)	0.0521 (8)	0.2613 (19)
H5AA	0.953206	0.265798	0.669311	0.078*	0.2613 (19)
H5AB	0.855187	0.304251	0.746069	0.078*	0.2613 (19)
H5AC	0.924122	0.370407	0.676741	0.078*	0.2613 (19)
C6A	0.3201 (9)	0.0845 (5)	0.6622 (5)	0.0470 (6)	0.2613 (19)
H6AA	0.303480	0.070051	0.601507	0.056*	0.2613 (19)
H6AB	0.245523	0.129747	0.679118	0.056*	0.2613 (19)
C7A	0.299 (3)	0.0026 (12)	0.7147 (16)	0.0514 (10)	0.2613 (19)
H7AA	0.272633	−0.047288	0.677574	0.077*	0.2613 (19)
H7AB	0.218138	0.012506	0.755778	0.077*	0.2613 (19)
H7AC	0.391725	−0.010925	0.745083	0.077*	0.2613 (19)
H1	0.871 (2)	0.1397 (13)	0.4883 (11)	0.038 (5)*	

### Diethyl [({2-[2-(diethoxyphosphinoyl)ethanamido]ethyl}carbamoyl)methyl]phosphonate (II) Atomic displacement parameters (Å^2^)

**Table:**

	*U*^11^	*U*^22^	*U*^33^	*U*^12^	*U*^13^	*U*^23^
P1	0.0313 (3)	0.0177 (4)	0.0384 (4)	0.0034 (3)	0.0024 (3)	0.0043 (3)
O2	0.0366 (11)	0.0231 (11)	0.0610 (14)	0.0061 (8)	0.0015 (8)	0.0068 (9)
O3	0.0456 (14)	0.0265 (12)	0.0372 (9)	0.0008 (8)	−0.0094 (9)	0.0011 (7)
O4	0.0430 (8)	0.0243 (8)	0.0409 (9)	0.0049 (6)	0.0152 (7)	0.0033 (6)
C2	0.0263 (9)	0.0256 (12)	0.0248 (18)	0.0006 (8)	0.0027 (13)	0.0054 (13)
C4	0.052 (2)	0.0284 (12)	0.0494 (10)	0.0007 (15)	−0.0097 (18)	0.0029 (9)
C5	0.053 (3)	0.0453 (18)	0.0576 (14)	−0.0050 (17)	−0.012 (2)	0.0038 (12)
C6	0.0605 (13)	0.0392 (11)	0.0413 (14)	−0.0010 (10)	0.0244 (14)	−0.0052 (12)
C7	0.054 (3)	0.0554 (12)	0.045 (2)	−0.0040 (14)	0.0188 (16)	0.0094 (12)
O1	0.0330 (5)	0.0251 (5)	0.0526 (6)	−0.0028 (4)	−0.0023 (5)	0.0110 (5)
N1	0.0263 (6)	0.0241 (6)	0.0496 (8)	−0.0009 (5)	0.0012 (5)	0.0071 (6)
C1	0.0285 (7)	0.0231 (7)	0.0343 (7)	−0.0023 (5)	−0.0019 (5)	0.0030 (5)
C3	0.0257 (7)	0.0333 (8)	0.0453 (8)	0.0002 (6)	−0.0029 (6)	0.0010 (7)
P1A	0.0313 (3)	0.0177 (4)	0.0384 (4)	0.0034 (3)	0.0024 (3)	0.0043 (3)
O2A	0.0366 (11)	0.0231 (11)	0.0610 (14)	0.0061 (8)	0.0015 (8)	0.0068 (9)
O3A	0.0456 (14)	0.0265 (12)	0.0372 (9)	0.0008 (8)	−0.0094 (9)	0.0011 (7)
O4A	0.0430 (8)	0.0243 (8)	0.0409 (9)	0.0049 (6)	0.0152 (7)	0.0033 (6)
C2A	0.0263 (9)	0.0256 (12)	0.0248 (18)	0.0006 (8)	0.0027 (13)	0.0054 (13)
C4A	0.052 (2)	0.0284 (12)	0.0494 (10)	0.0007 (15)	−0.0097 (18)	0.0029 (9)
C5A	0.053 (3)	0.0453 (18)	0.0576 (14)	−0.0050 (17)	−0.012 (2)	0.0038 (12)
C6A	0.0605 (13)	0.0392 (11)	0.0413 (14)	−0.0010 (10)	0.0244 (14)	−0.0052 (12)
C7A	0.054 (3)	0.0554 (12)	0.045 (2)	−0.0040 (14)	0.0188 (16)	0.0094 (12)

### Diethyl [({2-[2-(diethoxyphosphinoyl)ethanamido]ethyl}carbamoyl)methyl]phosphonate (II) Geometric parameters (Å, º)

**Table:**

P1—O2	1.474 (2)	C1—C2A	1.580 (15)
P1—O3	1.5791 (16)	C3—C3^i^	1.523 (3)
P1—O4	1.5619 (15)	C3—H3A	0.9900
P1—C2	1.801 (2)	C3—H3B	0.9900
O3—C4	1.464 (3)	P1A—O2A	1.473 (7)
O4—C6	1.458 (3)	P1A—O3A	1.570 (6)
C2—H2A	0.9900	P1A—O4A	1.581 (5)
C2—H2B	0.9900	P1A—C2A	1.760 (9)
C2—C1	1.501 (5)	O3A—C4A	1.435 (10)
C4—H4A	0.9900	O4A—C6A	1.413 (9)
C4—H4B	0.9900	C2A—H2AA	0.9900
C4—C5	1.502 (4)	C2A—H2AB	0.9900
C5—H5A	0.9800	C4A—H4AA	0.9900
C5—H5B	0.9800	C4A—H4AB	0.9900
C5—H5C	0.9800	C4A—C5A	1.489 (10)
C6—H6A	0.9900	C5A—H5AA	0.9800
C6—H6B	0.9900	C5A—H5AB	0.9800
C6—C7	1.475 (6)	C5A—H5AC	0.9800
C7—H7A	0.9800	C6A—H6AA	0.9900
C7—H7B	0.9800	C6A—H6AB	0.9900
C7—H7C	0.9800	C6A—C7A	1.495 (12)
O1—C1	1.2282 (17)	C7A—H7AA	0.9800
N1—C1	1.3348 (18)	C7A—H7AB	0.9800
N1—C3	1.4502 (19)	C7A—H7AC	0.9800
N1—H1	0.832 (19)		
			
O2—P1—O3	113.34 (11)	N1—C1—C2A	117.1 (6)
O2—P1—O4	115.38 (11)	N1—C3—C3^i^	111.71 (16)
O2—P1—C2	113.46 (18)	N1—C3—H3A	109.3
O3—P1—C2	106.70 (17)	N1—C3—H3B	109.3
O4—P1—O3	103.93 (11)	C3^i^—C3—H3A	109.3
O4—P1—C2	102.95 (16)	C3^i^—C3—H3B	109.3
C4—O3—P1	118.74 (19)	H3A—C3—H3B	107.9
C6—O4—P1	121.77 (14)	O2A—P1A—O3A	117.5 (4)
P1—C2—H2A	109.5	O2A—P1A—O4A	115.7 (4)
P1—C2—H2B	109.5	O2A—P1A—C2A	110.4 (6)
H2A—C2—H2B	108.0	O3A—P1A—O4A	97.9 (4)
C1—C2—P1	110.9 (2)	O3A—P1A—C2A	108.6 (6)
C1—C2—H2A	109.5	O4A—P1A—C2A	105.6 (5)
C1—C2—H2B	109.5	C4A—O3A—P1A	119.8 (7)
O3—C4—H4A	110.0	C6A—O4A—P1A	119.0 (5)
O3—C4—H4B	110.0	C1—C2A—P1A	121.1 (8)
O3—C4—C5	108.4 (3)	C1—C2A—H2AA	107.1
H4A—C4—H4B	108.4	C1—C2A—H2AB	107.1
C5—C4—H4A	110.0	P1A—C2A—H2AA	107.1
C5—C4—H4B	110.0	P1A—C2A—H2AB	107.1
C4—C5—H5A	109.5	H2AA—C2A—H2AB	106.8
C4—C5—H5B	109.5	O3A—C4A—H4AA	109.9
C4—C5—H5C	109.5	O3A—C4A—H4AB	109.9
H5A—C5—H5B	109.5	O3A—C4A—C5A	109.1 (9)
H5A—C5—H5C	109.5	H4AA—C4A—H4AB	108.3
H5B—C5—H5C	109.5	C5A—C4A—H4AA	109.9
O4—C6—H6A	109.9	C5A—C4A—H4AB	109.9
O4—C6—H6B	109.9	C4A—C5A—H5AA	109.5
O4—C6—C7	108.9 (3)	C4A—C5A—H5AB	109.5
H6A—C6—H6B	108.3	C4A—C5A—H5AC	109.5
C7—C6—H6A	109.9	H5AA—C5A—H5AB	109.5
C7—C6—H6B	109.9	H5AA—C5A—H5AC	109.5
C6—C7—H7A	109.5	H5AB—C5A—H5AC	109.5
C6—C7—H7B	109.5	O4A—C6A—H6AA	109.4
C6—C7—H7C	109.5	O4A—C6A—H6AB	109.4
H7A—C7—H7B	109.5	O4A—C6A—C7A	111.1 (13)
H7A—C7—H7C	109.5	H6AA—C6A—H6AB	108.0
H7B—C7—H7C	109.5	C7A—C6A—H6AA	109.4
C1—N1—C3	120.79 (12)	C7A—C6A—H6AB	109.4
C1—N1—H1	119.9 (13)	C6A—C7A—H7AA	109.5
C3—N1—H1	119.3 (13)	C6A—C7A—H7AB	109.5
O1—C1—C2	122.4 (2)	C6A—C7A—H7AC	109.5
O1—C1—N1	122.64 (13)	H7AA—C7A—H7AB	109.5
O1—C1—C2A	119.3 (6)	H7AA—C7A—H7AC	109.5
N1—C1—C2	114.9 (2)	H7AB—C7A—H7AC	109.5
			
P1—O3—C4—C5	−160.9 (3)	C1—N1—C3—C3^i^	77.0 (2)
P1—O4—C6—C7	170.3 (4)	C3—N1—C1—C2	−176.50 (16)
P1—C2—C1—O1	71.4 (3)	C3—N1—C1—O1	0.4 (2)
P1—C2—C1—N1	−111.8 (2)	C3—N1—C1—C2A	169.1 (4)
O2—P1—O3—C4	−45.5 (3)	P1A—O3A—C4A—C5A	−172.5 (10)
O2—P1—O4—C6	−48.7 (2)	P1A—O4A—C6A—C7A	165.3 (11)
O2—P1—C2—C1	174.3 (2)	O2A—P1A—O3A—C4A	−44.3 (11)
O3—P1—O4—C6	76.0 (2)	O2A—P1A—O4A—C6A	40.5 (6)
O3—P1—C2—C1	48.8 (3)	O2A—P1A—C2A—C1	172.8 (8)
O4—P1—O3—C4	−171.6 (2)	O3A—P1A—O4A—C6A	166.2 (6)
O4—P1—C2—C1	−60.3 (3)	O3A—P1A—C2A—C1	42.7 (11)
C2—P1—O3—C4	80.1 (3)	O4A—P1A—O3A—C4A	−168.7 (8)
C2—P1—O4—C6	−172.9 (2)	O4A—P1A—C2A—C1	−61.4 (11)
O1—C1—C2A—P1A	76.3 (10)	C2A—P1A—O3A—C4A	81.9 (10)
N1—C1—C2A—P1A	−92.8 (9)	C2A—P1A—O4A—C6A	−81.9 (7)

Symmetry code: (i) −*x*+2, −*y*, −*z*+1.

### Diethyl [({2-[2-(diethoxyphosphinoyl)ethanamido]ethyl}carbamoyl)methyl]phosphonate (II) Hydrogen-bond geometry (Å, º)

**Table:**

*D*—H···*A*	*D*—H	H···*A*	*D*···*A*	*D*—H···*A*
N1—H1···O2^ii^	0.832 (19)	2.05 (2)	2.883 (2)	175.0 (18)
N1—H1···O2*A*^ii^	0.832 (19)	1.92 (2)	2.741 (8)	170.2 (18)

Symmetry code: (ii) *x*+1/2, −*y*+1/2, −*z*+1.
